# Supplementary material for: Self-efficacy and career intention: Uncovering the underlying processes
Source: PLoS One. 2025 Jan 3;20(1):e0314802. doi: 10.1371/journal.pone.0314802 (PMC11698408; doi:10.1371/journal.pone.0314802)
Supplement: Attachment — Submitted filename: review.docx [file pone.0314802.s003.docx]

**Introduction**

Provide a source for the first sentence. Again, the researchers failed to buttress most claims with literature.

While the study draws on the Theory of Planned Behavior (TPB), there is insufficient explanation of how TPB specifically relates to P-E fit and career attitudes in this context. This could leave the reader questioning the theoretical integration.

There is a lack of consistent definitions for some key terms. For instance, "career intentions" and "career attitudes" are mentioned multiple times, but their distinct meanings and interrelationships aren't always clear.

Although the passage mentions the scarcity of research in South Asian contexts, particularly Bangladesh, it does not sufficiently discuss why this cultural setting is significant for understanding the relationship between self-efficacy and career intentions.

The passage does not clearly justify why a theoretical model with sequential mediation is the most appropriate approach for investigating the relationships in question.

The introduction lacks coordination and its difficult to understand what the authors want to achieve. Kindly reorganize your thoughts and improve on it.

**Literature Review and Hypotheses Development**

There is a misfit here, in the introduction, the researchers provided that TPB has been overly used and so they proposed a theory, however, this theory has not been addressed. Kindly start the argument of the hypothesis development with the theory. Again, is the P-E fit a theory or what? Kindly be specific.

**Methods**

The researcher did well by stating the time period for data collection, however, don’t you think that the issue the researchers are attending to has been addressed or changed looking at the time duration. What was the target population and what was the actual sample size before distributing the instrument.

Why did the researchers mention control variables in this section but did not discuss their role and implication in the introduction?

**Discussion of Results**

Kindly discuss the results in lune with the theory.
