# Supplementary material for: Self-efficacy and career intention: Uncovering the underlying processes
Source: PLoS One. 2025 Jan 3;20(1):e0314802. doi: 10.1371/journal.pone.0314802 (PMC11698408; doi:10.1371/journal.pone.0314802)
Supplement: Attachment — Submitted filename: Response to Reviewers.docx [file pone.0314802.s004.docx]

Manuscript Title:

**Self-efficacy and Career Intention: Uncovering the Underlying Processes**

(Manuscript ID: PONE-D-24-25408.R1)

First of all, we take the opportunity to thank the well-valued journal and its dedicated editor for allowing us to submit the revised manuscript. We also would like to thank the respected editor and reviewer for their worthy comments. The comments have indeed helped us to improve the quality of the paper. We have tried to improve each and every section of our manuscript to make it more convincing. We are open to any further improvements in this paper. Please find below our responses to the comments made by the editor and reviewers.

| **EDITOR** | |
| --- | --- |
| **Editor’s Comments** | **Authors’ Response** |
| Thank you for submitting your manuscript to PLOS ONE. After careful consideration, we feel that it has merit but does not fully meet PLOS ONE’s publication criteria as it currently stands. Therefore, we invite you to submit a revised version of the manuscript that addresses the points raised during the review process. | Thank you for allowing us to revise and resubmit our manuscript. We are humbled that our manuscript has been well received and found as a potential for publication in the journal. We have addressed all the comments on a point-by-point basis. In fact, a detailed effort has been made to improve each and every part of our manuscript with recent publications to make it more convincing to our potential readers. |
| **REVIEWER 1** | |
| **Reviewer’s Comments** | **Authors’ Response** |
| 1. Can you provide more detailed definitions of the key constructs (career self-efficacy, P-E fit, career attitudes, and career intentions) to ensure clarity for readers who may not be familiar with these concepts? | 1. Thank you for your valuable feedback. As per your suggestion, we have further updated the definition of our study variables with more recent citations. For details, please see the highlighted parts under the section 2.1 of our revised manuscript. |
| 2. How was the sample of 341 job seekers in Bangladesh selected, and does this sample adequately represent the broader population? Could you discuss any potential sampling biases and how they might impact the generalizability of the findings? | 2. Thank you. As you suggest, we have highlighted the sampling procedure in the “Participants and Procedure” section of our revised manuscript. However, as the concern arouse, we have incorporated your thoughts into the limitation section also. For further information, please see highlighted parts under the section 3.1 and 5.3 of our revised work.  The sample size (N = 341) of our study adequately represents the broader population, as it exceeded the minimum sample size requirement for the four focal constructs, that is, N > 50 + 8m, where m = number of predictors (Pallant, 2016; Tabachnick & Fidell, 2013). For details, please refer to the following publications:  Pallant, J. (2016). *SPSS Survival Manual: A Step by Step Guide to Data Analysis Using IBM SPSS*. McGraw-Hill Education.  Tabachnick, B. G., & Fidell, L. S. (2013). *Using Multivariate Statistics*. Pearson Education Inc. |
| 3. Could you elaborate on the specific procedures used in AMOS and SPSS Process Macro? For instance, how were the mediation effects tested, and were any assumptions made during the analysis that could influence the results? | Thank you for your comments. To check the common method bias (CMB), composite reliability, convergent and discriminant validity of our study variables, we performed measurement model analysis through AMOS. Besides, to test the simple mediation analysis, we developed path model through the AMOS. For details, please see section 4.1 and 4.2 of our revised manuscript.  However, to confirm the sequential mediation, we used PROCESS Macro as it is considered as a more powerful statistical tool for probing mediation. To test the sequential mediation hypothesis, we directly examined the significance of indirect effects of career self-efficacy on career intentions through serial multiple mediators. We analyzed the data with 5000 bootstrap samples of 95% bias-corrected confidence intervals (CI) using Model 6, as recommended in Hayes’s PROCESS Macro. For details, please see section 4.3 of our revised manuscript.  It is important to note that we also checked the CB-SEM assumptions (i.e., linearity, normality, outliers, multicollinearity, and homoscedasticity) before testing the research hypotheses. A test of normal probability plot of residuals, frequency of regression residuals, and the scatterplot of residuals against predicted values revealed that the regressions assumptions were met. |
| 4. How were the measurement tools (e.g., scales for career self-efficacy, P-E fit, and career attitudes) validated in this study? Please attached instrument with details source. | 4. Thank you. As per your suggestions, we have highlighted how we confirmed the validity of our study constructs. We have also attached the measurement items at the end of our revised manuscript. For details, please see our revised work. |
| 5. The study suggests that P-E fit and career attitudes sequentially mediate the relationship between career self-efficacy and career intentions. Could you provide a more in-depth discussion on why this sequence was hypothesized and how it aligns with existing literature? | 5. Thank you for bringing this point to our concentration. As you suggest, we have further highlighted the arguments for hypothesizing the sequential mediating effects [please see section 2.5]. In addition, in our “Introduction” section, we have noted why the sequential mediation is necessary and how it aligns with existing literature. For further information, please also see the highlighted part of the last paragraph under the “Introduction” section of our revised work. |
| 6. The paper mentions notable implications for career counselors and HR managers. Could you expand on these practical implications, possibly providing examples of how the findings can be applied in real-world settings? | 6. Thank you. As per your suggestions, we have included some examples of how our study findings can be applied in real-world settings. For details, please see the highlighted parts under the “Managerial Implications” section of our revised manuscript. |
| 7. While the study discusses the implications of the findings, could you also elaborate on the limitations of your study and suggest areas for future research to build upon your findings? | 7. Thank you. As you suggest, we have further updated the “Limitations and Future Research Directions” section of our revised work. For details, please see the section. |
| 8. Given that the study was conducted in Bangladesh, how might cultural factors influence the relationships among the constructs? Would you consider discussing how these findings could be generalized to other cultural contexts or whether cultural differences might alter the results? | 8. Thank you for the comments. If we are not wrong, we believe that we have already addressed this concern through addressing your previous comment 8. For details, please see the last paragraph of the “Limitations and Future Research Directions” section of our revised work. |
| 9. In revision version please provide dataset for check statistical measures. | 9. Thank you. We have included our original dataset. |
| **REVIEWER 2** | |
| **Reviewer’s Comments** | **Authors’ Response** |
| *Introduction*  Provide a source for the first sentence. Again, the researchers failed to buttress most claims with literature. | We appreciate your comment. We have updated the sentence with citation. Please see our revised manuscript. |
| While the study draws on the Theory of Planned Behavior (TPB), there is insufficient explanation of how TPB specifically relates to P-E fit and career attitudes in this context. This could leave the reader questioning the theoretical integration. | Thank you bringing this point to our attention. We have updated our work with two new sections where we have discussed why the integration of P-E fit with the TPB is necessary under the present study settings. For further information, please see the highlighted parts under the section 2.1 of our revised manuscript. |
| There is a lack of consistent definitions for some key terms. For instance, "career intentions" and "career attitudes" are mentioned multiple times, but their distinct meanings and interrelationships aren't always clear. | Thank you. As per your suggestion, we have further updated the definition of our study variables with more recent citations. For details, please see the highlighted parts under the section 2.1 of our revised manuscript. |
| Although the passage mentions the scarcity of research in South Asian contexts, particularly Bangladesh, it does not sufficiently discuss why this cultural setting is significant for understanding the relationship between self-efficacy and career intentions. | Thank you for pointing this issue to our mind. As per your observation, we have developed a better case to justify why the cultural setting is significant for understanding the relationship between self-efficacy and career intentions with relevant citation from scholarly journals. For details, please see highlighted parts of the first paragraph under the “Introduction” section of our revised manuscript. |
| The passage does not clearly justify why a theoretical model with sequential mediation is the most appropriate approach for investigating the relationships in question. | Thank you for your valuable feedback. As the concern arouse, we have developed a better case to show why a theoretical model with sequential mediation is the most appropriate approach for investigating the relationships in question under the “Introduction” section [please see the last paragraph of the section]. In addition, we also discussed it while proposing the hypothesis (H3) related to sequential mediation to follow the chain of arguments. For details, we request you have a look at the section 2.5 of our revised work. |
| The introduction lacks coordination and its difficult to understand what the authors want to achieve. Kindly reorganize your thoughts and improve on it. | Thank you for your comments. Please note that we have reviewed more literature in the related field and developed a better case to further justify the rationale of our study with more recent citations. For details, please see the “Introduction” section of our revised manuscript. We hope that you will find it satisfactory. |
| *Literature Review and Hypotheses Development*  There is a misfit here, in the introduction, the researchers provided that TPB has been overly used and so they proposed a theory, however, this theory has not been addressed. Kindly start the argument of the hypothesis development with the theory. Again, is the P-E fit a theory or what? Kindly be specific. | We appreciate your observations here. As per your suggestion, we have updated our paper with two new sections “Theory of Planned Behavior” and “Person-Environment (P-E) Fit” to show their applicability to our research model. We utilized P-E fit as a concept stemmed from Fit Theory. For further information, please the section 2.1 and 2.2 of our revised version of work. |
| *Methods*  The researcher did well by stating the time period for data collection, however, don’t you think that the issue the researchers are attending to has been addressed or changed looking at the time duration. What was the target population and what was the actual sample size before distributing the instrument. | Thank you for your valuable feedback. We appreciate the opportunity to clarify these points.  **Relevance of the study over time:** While the data collection period was mentioned, we acknowledge your concern regarding whether the issue being studied may have changed over time. It is important to highlight that the core focus of the study—career self-efficacy, P-E fit perceptions, career attitudes, and career intentions—remains relevant, as these variables are influenced by enduring psychological processes rooted in established theories such as the theory of planned behavior. Although some external factors, such as market conditions or organizational practices, may evolve over time, the fundamental relationships between these variables are unlikely to change significantly in a short duration.  **Target population:** The target population for this study consisted of prospective job seekers and university graduates. This group was chosen because they were actively engaged in career-related decision-making, making them suitable for examining the research variables related to self-efficacy, P-E fit, career attitudes, and intentions.  **Actual sample size before distributing the instrument:** Before distributing the instrument, the initial target sample size was 500 participants. This estimate was based on statistical power calculations to ensure adequate representation. After distributing the instrument, we ultimately achieved a final sample size of 341. This sample size was sufficient for conducting the planned statistical analyses and provided a robust dataset for drawing meaningful conclusions. |
| Why did the researchers mention control variables in this section but did not discuss their role and implication in the introduction? | Thank you for pointing this issue to our attention. Please note that following the past studies in this field, we have controlled demographic profiles of our respondents. But the preliminary analysis reported no significant effect. That’s why did not discuss their role and implication. However, as the concern arouse, we acknowledge it into the limitation section of our paper. For details, please see the last paragraph of limitation section of our revised paper. |
| *Discussion of Results*  Kindly discuss the results in line with the theory. | Thank you for your comments. As the concern arouse, we have read the section thoroughly and removed any sentences if it was found inconsistent with our study findings. For details, please see the highlighted parts of the “Discussion” section of our revised our work. |

With regards,

The authors
